# Connexive Exclusion

**DOI:** 10.1007/s10670-024-00842-3

**Published:** 2024-08-27

**Authors:** Yaroslav Shramko, Heinrich Wansing

**Affiliations:** 1 Department of Philosophy, Kryvyi Rih State Pedagogical University, Kryvyi Rih, 50086 Ukraine; 2 Department of Philosophy I, Ruhr University Bochum, 44780 Bochum, Germany

## Abstract

We present a logic which deals with connexive exclusion. Exclusion (also called “co-implication”) is considered to be a propositional connective dual to the connective of implication. Similarly to implication, exclusion turns out to be non-connexive in both classical and intuitionistic logics, in the sense that it does not satisfy certain principles that express such connexivity. We formulate these principles for connexive exclusion, which are in some sense dual to the well-known Aristotle’s and Boethius’ theses for connexive implication. A logical system in a language containing exclusion and negation can be called a logic of connexive exclusion if and only if it obeys these principles, and, in addition, the connective of exclusion in it is asymmetric, thus being different from a simple mutual incompatibility of propositions. We will develop a certain approach to such a logic of connexive exclusion based on a semantic justification of the connective in question. Our paradigm logic of connexive implication will be the connexive logic ${\textsf{C}}$, and exactly like this logic the logic of connexive exclusion turns out to be contradictory though not trivial.

## Introduction: Implication and Exclusion

According to Storrs McCall (1975, p. 435), “[c]onnexive logic represents an attempt to formalize the species of implication recommended by Chrysippus:

And those who introduce the notion of connexion say that a conditional is sound when the contradictory of its consequent is incompatible with its antecedent. (Sextus Empiricus, translated in (Kneale & Kneale, 1962, p. 129)).”

Under this (ancient) approach implication (“the heart of logic”, as Anderson and Belnap (1975, p. 3) once put it) turns out to be a definable notion being introduced in terms of the even more fundamental notion of (in)compatibility. Now, if $\rightarrow$, $\circ$ and $\sim$ stand for the connectives of implication, compatibility and negation respectively, one obtains Chrysippus’ definition of implication formulated by McCall as follows (ibid.):

### Definition 1

$$A\rightarrow B =_{df} {\sim }(A \circ {\sim } B)$$.

Pizzi (2004, p. 561) draws attention to a paper of Everett Nelson (1930), in which essentially the same definition is given, with $\circ$ being interpreted as consistency or compossibility. Pizzi himself prefers to speak of “cotenability”—the term “introduced by Nelson Goodman (1947) in the context of the analysis of counterfactuals” (ibid.).^1^ He then defines “the abstract notion of implication” (*Imp*) as “the dual of cotenability” (*Cot*):

### Definition 2

$$A~{\textit{Imp} }~B =_{df} ~{\sim }(A~{ \textit{Cot} }~{\sim } B)$$.

Thus, the idea of introducing (and explaining) implication through another fundamental notion, such as (in)consistency, (in)compatibility or (non-)cotenability, not only has deep philosophical roots, but has also been recently pursued in the literature. Remarkably, the relations of consistency, compatibility and cotenability are all essentially symmetric, so that, e.g., if *A* is (in)consistent with *B*, then *B* also is (in)consistent with *A*. At the same time implication is generally non-symmetric, and it is overall possible that *A* implies *B*, while *B* does not imply *A*. It seems therefore only natural if a fundamental relation used to interpret implication would also be non-symmetric.

A good candidate for such a non-symmetric relation could be represented by the notion of (directed) *exclusion*. Such a relation will differ from, e.g., simple inconsistency in that one situation could exclude another situation without being excluded by it. Then Chrysippus’ view on implication could be reformulated in such a way that a conditional is considered to be sound when its antecedent *excludes* the contradictory of its consequent. In what follows we will argue that the notion of exclusion so understood is in a certain sense dual to implication. Let *Exc* stands for such a notion. Then we can have another version of Chrysippus’s definition of implication:

### Definition 3

$$A~{ \textit{Imp} }~B =_{df}~A~{ \textit{Exc} }~{\sim } B$$.^2^

Another issue related to the idea of implication in the spirit of Chrysippus is *connexivity*. The elaboration of this issue has led to the emergence of a whole branch of modern non-classical logic—connexive logic, which deals with the notion of connexive implication. Again, it seems quite natural that exclusion, which could be dual to connexive implication, should also be connexive. Hence, there arises the task of constructing a special logic dealing with connexive exclusion.

In this paper, we will develop a certain approach to such a logic of connexive exclusion in various versions. Section 2 will introduce the connective of connexive exclusion, and present some requirements for a logic dealing with this connective. In Sect. 3 we will propose a semantical justification of the connective in question. In Sect. 4 the proposed semantics of connexive exclusion will be deductively formalized by a (restricted) consequence proof system. In Sect. 5 a sound and complete sequent system for the logic of connexive exclusion will be constructed, and Sect. 6 will present some bi-connexive systems. The paper will end with some concluding remarks and an indication of possible future work.

## Exclusion and Connexivity

Among the propositional connectives that are found in various logical systems, there are some that can be called (and often are indeed called) “traditional”, “standard” or “common”, because they are almost always (or at least very often) used in the construction of all these systems. Here we mean primarily such connectives as *conjunction*, *disjunction*, *implication* (also called *conditional*) and *negation* of various kinds. Indeed, it is difficult to find a logical system with sophisticated expressiveness where these connectives would not be employed. The Britannica (2007) in the entry “Connective (logic)” presents exactly these connectives as “commonly used” by explaining that “various types of logical connectives include conjunction (“and”), disjunction (“or”), negation (“not”), conditional (“if ...then”), and biconditional (“if and only if”).” (Note that a biconditional is a straightforward conjunction of two conditionals.) And Humberstone’s seminal monograph (Humberstone, 2011), a comprehensive and in-depth study of connectives, devotes separate chapters to *And*, *Or*, *If*, and *Not*. But of course we know that there can be many other logical operators. For example, the number of binary Boolean operations is 16, and if we further think of intensional logical connectives which can be modeled, say, with the help of Kripke structures, there may be infinitely many of them. Alas, for many such connectives it is very difficult to find suitable correspondences in natural language, which is maybe why they rarely go beyond specialized technical texts on logic and mathematics.

But here we want to focus on a logical connective which is often neglected, undeservedly we believe, although it may have a quite natural justification from the point of view of natural language—the connective dual to implication. In classical logic, dual connectives are obtained from each other by interchanging between values 1 (true) and 0 (false) in their truth table definitions, see, e.g., (Church, 1956, pp. 106–108). Thus, the connective dual to the classical (material) implication, denoted by $\not \subset$, obtains the definition presented in Table 1. From this table we can see, incidentally, that material dual implication is equivalent to the negation of converse material implication, so Church (1956, p. 37) calls this connective “converse non-implication” (whence the corresponding symbolism), giving it the reading “not..., but...”. This is not surprising given the mutual duality of classical disjunction and conjunction, and self-duality of classical negation: since the material implication “if *A*, then *B*” is equivalent to “not-*A* or *B*”, the material dual implication turns out to be equivalent to “not-*A* and *B*”, or “it is not the case that if *B*, then *A*”.

**Table 1:** Classical (material) dual implication

*A*	*B*	$A \not \subset B$
1	1	0
1	0	0
0	1	1
0	0	0

However, in the contexts of non-classical logics, a connective dual to implication can receive a more refined interpretation. One such context is provided by intuitionistic logic, the dual counterpart of which (known as dual intuitionistic logic) provides a suitable framework for defining the dual intuitionistic implication. We can consider this framework from a semantic standpoint by constructing suitable Kripke models.

A Kripke frame is a pair $\mathfrak {F} = \langle W, \le \rangle$, where *W* is a non-empty set of states, and $\le$ is a reflexive and transitive (“accessibility”) relation on *W*. Then an intuitionistic I-model based on a Kripke frame $\mathfrak {F}$ is a structure $\mathfrak {M}^I = \langle W, {\le ,} v^+, v^-\rangle$, where $v^+$ and $v^-$ are valuation functions from the set of atomic formulas (propositional variables) into $\mathcal {P}(W)$, the powerset of *W*, subject to the following conditions for every *p* and ${\alpha }\in W$: 
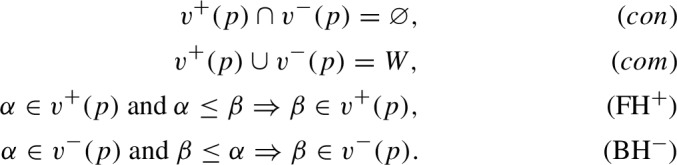


Intuitively, $v^+(p)$ is the set of states that supports the truth of *p*, and $v^-(p)$ is the set of states that supports the falsity of *p*. The conditions (*con*) and (*com*) ensure that there are no valuation gluts and gaps, whereas ((FH$^+$)) and (BH$^-$) secure, respectively, forward and backward persistency (hereditariness) along $\le$.

Consider the language $\mathcal{L}^I$ of intuitionistic propositional logic defined as follows:^3^

$$\begin{aligned} A {:}{:} = p \mid (A\wedge A) \mid (A \vee A) \mid (A \succ A) \mid \lnot A. \end{aligned}$$

In each I-model $\mathfrak {M}^I$ the relations ${\mathfrak {M}^I,} {\alpha }\models ^+ A$ (${\alpha }$ supports the truth of *A* in $\mathfrak {M}^I$) and ${\mathfrak {M}^I,} {\alpha }\models ^- A$ (${\alpha }$ supports the falsity of *A* in $\mathfrak {M}^I$) are inductively defined as follows (where ${\alpha }, {\beta }\in W$): 
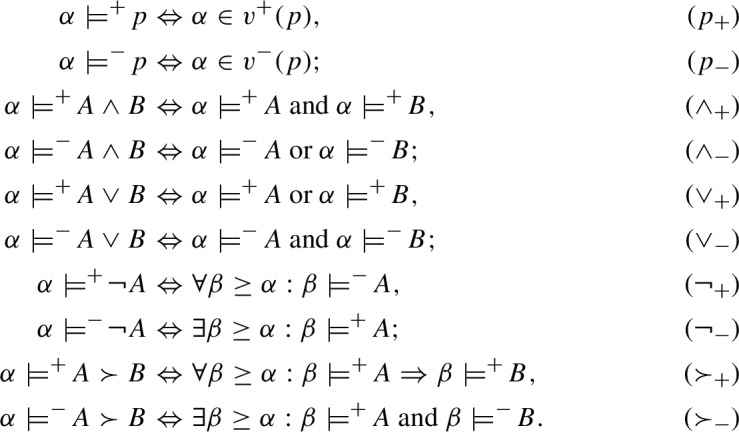


Here we have omitted, for the sake of brevity, the indication of the model, just writing ${\alpha }\models ^+\! A$ and ${\alpha }\models ^-\! A$ instead of $\mathfrak {M}^I, {\alpha }\models ^+\! A$ and $\mathfrak {M}^I, {\alpha }\models ^-\! A$. Hereafter, we will continue to use this abbreviated mode of expression, unless the reference to the model is required for the purposes of the presentation.

By induction, (FH$^+$) and (BH$^-$) can be extended to any formula *A*. Validity of a formula in a model, intuitionistic validity and the entailment relation are defined as usual:

### Definition 4

1. $$\models _{\mathfrak {M}^I}\!A =_{df} \forall {\alpha }\in W: {\mathfrak {M}^I,} {\alpha }\models ^+\! A;$$
2. $$\models _{\textsf{I}}\!A =_{df} \forall \,\mathfrak {M}^I \!\models _{\mathfrak {M}^I}\!A;$$
3. $$\Gamma \models _{\mathfrak {M}^I}\!A =_{df} \forall {\alpha }\in W (\forall B\in \Gamma : {\mathfrak {M}^I,} {\alpha }\models ^+\! B) \Rightarrow {\mathfrak {M}^I,} {\alpha }\models ^+\! A;$$
4. $${\Gamma }\models _{\textsf{I}}\!A =_{df} \forall \,\mathfrak {M}^I: {\Gamma }\models _{\mathfrak {M}^I}\!A$$.

### Remark 1

Note that a model for intuitionistic logic can be, and usually is, equivalently defined to be a structure $\mathfrak {M}^I = \langle W, \le , v\rangle$, where $\langle W, \le \rangle$ is a Kripke frame and *v* is a valuation function from the set of atomic formulas into $\mathcal {P}(W)$ satisfying (FH$^+$). Then a single verification relation, $\models$, between states and $\mathcal{L}^I$-formulas is defined by replacing ‘$\models ^+$’ by ‘$\models$’ in the above definition. Due to the conditions (*con*) and (*com*), if one is given a model $\mathfrak {M}^I = \langle W, \le , v\rangle$, valuation functions $v^+$ and $v^-$ can be defined by setting $v^+(p):= v(p)$, $v^-(p):= W\setminus v(p)$, and the support of truth and support of falsity relations can be defined by setting $\alpha \models ^+ A$ $:\Leftrightarrow$ $\alpha \models A$ and $\alpha \models ^- A$ $:\Leftrightarrow$ $\alpha \not \models A$. We shall make use of this presentation of I-models in Sect. 5. However, the use of two valuation functions (to define separately truth support and falsity support) is crucial for the construction of models in which (*con*) and (*com*) do not generally hold. Such models are characteristic of certain logics in which truth and falsity are treated as independent (symmetric) notions, e.g., David Nelson’s logics of constructible falsity (Almukdad & Nelson, 1984; Nelson, 1949) or some systems of relevance logic. Some of the logics introduced in this paper will also be of this kind, so we consider already here I-models with two valuation functions to facilitate a natural transition to models of these further logics.

Now, to proceed to the dual intuitionistic logic we may consider a dual intuitionistic language $\mathcal{L}^{DI}$determined as follows:Here $\nprec$ and  stand for the connectives of dual intuitionistic implication (also called *co-implication*, see, e.g., (Wolter, 1998)) and dual intuitionistic negation. A dual intuitionistic DI-model $\mathfrak {M}^{DI}$ can be obtained from an intuitionistic model $\mathfrak {M}^I$ by taking the following conditions instead of (FH$^+$) and (BH$^-$): 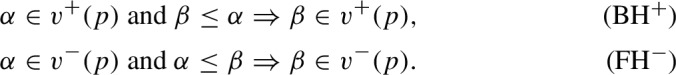


The relations $\models ^+$ and $\models ^-$ for $\nprec$ and  are defined by the following conditions: 
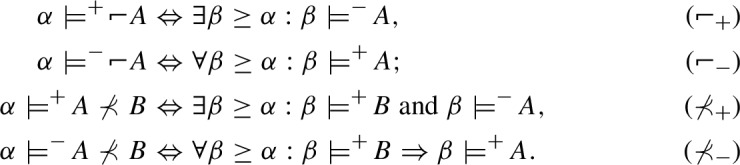


These conditions are obtained from the definitions of the corresponding intuitionistic connectives by simply interchanging between dual notions in the classical metalanguage used here ($\forall$ and $\exists$, as well as “and” and “or”). For example, the right-hand side of ($\succ _+$) can be equivalently rewritten as $\forall \beta \ge \alpha : {\beta }\models ^-\! A \text{ or } {\beta }\models ^+\! B$, from which we immediately obtain ($\nprec _+$) using the dualization procedure just described.^4^

Again, (BH$^-$) and (FH$^-$) can be extended to any formula *A*. Definitions of dual intuitionistic validity and entailment are as in Definition 4 by replacing ${M}^{I}$ with ${M}^{DI}$.

Turning now to the issue of an intuitive interpretation of the dual intuitionistic implication (co-implication), we should first note that the simple identification with “not ..., but ...”, as in classical logic, is not applicable here. In the dual intuitionistic logic the formulas  are not equivalent (just as $A \succ B$ and $\lnot A \vee B$ are not equivalent in intuitionistic logic). Urbas (1996, p. 451) makes this observation and suggests to interpret dual implication as a kind of exclusion operator: “whereas *A* implies *B* just in case the logical content of *A* must include that of *B*, dually *A* excludes *B* just in case the logical content of *A* cannot include that of *B*.” Note again, that in the spirit of Definition ($\nprec _+$), the expression dual to “*A* implies *B*” should rather be “*B* excludes *A*” or “*A* is excluded by *B*”. But anyway, we find this suggestion more fortunate than an alternative tradition of identifying co-implication with the algebraic operation of subtraction or pseudo-difference, which is also quite common in the literature.

This latter tradition goes back at least to a work of Skolem (1919), where some calculus of classes with four operations is investigated, for which Skolem employs names (and symbols) borrowed from arithmetic: addition, multiplication, subtraction and division. The characteristic property linking addition and subtraction is $(a\le b + x) = (a-b\le x)$, just as the property linking multiplication and division is $(ax\le b) = (x \le \frac{b}{a})$, see (Skolem, 1919, Theorem 7). If we consider logical addition, multiplication and division as disjunction, conjunction and implication respectively, then, given the duality between conjunction and disjunction, subtraction could be considered a logical operation dual to implication. With this interpretation, and with the amendment that logical subtraction so understood is actually dual to the converse implication, see, e.g., (Curry, 1963, p. 144) and (Crolard, 2001, p. 160), it could be suggested to read $A \nprec B$ as “*A* minus *B*” or “*A* *without* *B*”, see (Crolard, 2001, p. 159). While this reading and corresponding interpretation is quite acceptable for the material dual implication, it may seem problematic in a more general setting.

Indeed, an important difference between such connectives as conjunction and disjunction, on the one hand, and implication, on the other, is that the former are of a “factual” character, while the latter is not. Conjunctive and disjunctive formulas state facts ($A \wedge B$ says that both *A* and *B* are the case, whereas $A \vee B$ says that at least one of them is the case). In contrast, an implication, in general, should merely express some *connection* (namely, conditional connection) between possible states of affairs, regardless of whether those states of affairs actually occur. Of course, if a conditional $A \rightarrow B$ turns out to be equivalent to the disjunctive proposition $\lnot A \vee B$ (as in classical logic), then it becomes factual too. But already in intuitionistic logic this equivalence does not hold. In the light of the faithful embedding of intuitionistic logic into the modal logic S4, the intuitionistic conditional is a strict material implication. In intuitionistic logic, the truth value of a conditional proposition in some state (in contrast to a disjunctive one in Kripke semantics, though not in Beth-semantics) is established by taking also into account other possible states accessible from the one in which the evaluation is made. Thus, generally, the statement “if it is raining, the road is wet” says nothing about a situation with rain now, nor about the state of the road at a given time, but simply asserts some (conditional) connection between these possible situations.

From symmetry considerations, it seems natural to assume that a connective dual to implication should not be factual either, and is used to express some connection between propositions dual to the one expressed by the conditional.

Apparently the operation of subtraction does not quite fit into this understanding, for “*A* without *B*” seems to assert explicitly the actual presence of *A* and the absence of *B*. Exclusion, by contrast, is devoid of this flavor of factuality: by claiming that *B* excludes *A*, or *A* is excluded by *B*, we presumably do not make any commitment to the actual existence or non-existence of either *A* or *B*. This claim means only that *A* cannot be the case at the same time as *B*, and hence, that *A* would not be the case in the presence of *B*. But whether *B* (and thus, *A*) by itself is the case remains open. All this gives reasons to treat the dual implication as an exclusion operator, where the proposition that corresponds to the consequent of the original implication excludes the proposition that acted there as the antecedent, and this is the view we will take in the present paper.

It is also noteworthy that the operation of exclusion, like the implication operation, is in general not symmetric. Indeed, if *A* implies *B*, then *B* can also, of course, imply *A* (in which case we have the biconditional of the two propositions), but this is not necessarily true in general. Likewise, if *A* excludes *B*, then of course *B* can exclude *A* (and then we get incompatibility between the two propositions), but this need not hold in the general case. That John turns on the functioning heating system in his office, for example, excludes that it is cold in his office, but that it is cold in his office does not exclude that John turns on the heating system in his office. Similarly, just as rain implies a wet road but not the other way around, it is possible that rain excludes John’s walk in the park, but the reverse is not true.

Thus, for the operation of exclusion, just as for implication, a certain (directed) *connection* (connexion) between its components is of primary importance. There is an interesting idea associated with the notion of connexivity as applied to implication, which leads to the conception of *connexive implication*. The idea is that in a sound conditional no proposition can imply or be implied by its own negation, see (McCall, 1966). More generally, if a proposition implies some (possibly other) proposition, then the first proposition cannot imply the negation of the second proposition. This has a clear intuitive appeal in terms of the coherence or connection between the premises and the conclusions of valid inferences or between the antecedent and the succedent (consequent) of valid implications (Wansing, 2023a).

Formally, this intuition is usually implemented by two theses (each in two versions), one called *Aristotle’s thesis* (AT) and the other called *Boethius’ thesis* (BT). Let $\rightarrow$ stand for a connective of implication subject to the idea of connexivity as expressed above, and $\sim$ be a negation operator. Aristotle’s thesis, which expresses the fundamental connexivist intuition that it is impossible for a sentence to imply or be implied by its own negation, is then formulated through the following pair of principles:
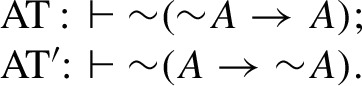


Boethius’ thesis holds that two statements, one saying that some proposition implies some (other) proposition, and the other saying that the first proposition implies the negation of that (other) proposition, are incompatible. Formally this is usually expressed by means of the following pair of principles: 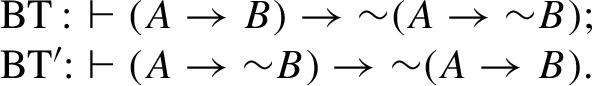


In this form BT and BT$^\prime$ are sometimes referred to as *strong* Boethius’ thesis, see, e.g., (Pizzi & Williamson, 1997). Also Boethius’ thesis can be expressed in a rule (consequence) form (in which it is sometimes called *Boethius’ rule* (Pizzi, 2005, p. 120)) as follows:
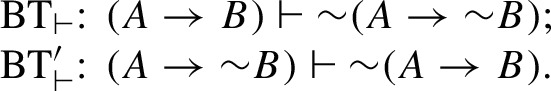


The general agreement is that *connexive logic* should validate AT, AT$^\prime$, BT, and BT$^\prime$, without the implication being symmetric, i.e., neither $\vdash (A\rightarrow B)\rightarrow (B\rightarrow A)$, nor $(A\rightarrow B)\vdash (B\rightarrow A)$ must hold. If Boethius’ thesis is taken in a rule form, the logic is sometimes called *weakly connexive*, see (Wansing & Unterhuber, 2019, p. 569), (Wansing & Omori, 2024). For a historical survey of the development of the idea of connexivity and connexive logic, see (McCall, 2012); for a terminological overview, see (Wansing & Omori, 2024). None of AT, AT$^\prime$, BT, or BT$^\prime$ are valid in classical logic, so any connexive logic should be contra-classical, see (Humberstone, 2000). There are a number of different systems of connexive logic, and one such system, the logic $\textsf{C}$, will be discussed in more detail in the next section.

Now, given the role of the connection between constituents not only in implicational propositions, but also in propositions with dual implication (exclusion), we can consider the idea of a *connexive exclusion*, which will be in some sense dual to the connexive implication. First of all, one might expect that a principle analogous to Aristotle’s thesis should apply to this kind of exclusion—any proposition must connexively exclude, and also be excluded by its own negation. This principle seems to be quite natural. Remarkably, in classical logic neither $-A\not \subset A$, nor $A \not \subset -A$ (where − is the Boolean negation) is valid, and hence the connective of material exclusion is not connexive in the sense just described. But also in dual intuitionistic logic we do not have connexivity for exclusion, since here again neither , nor  is valid. This actualizes the task of explicating the connective of exclusion from a connexivist perspective.

Let $\nleftarrow$ denote the connective of connexive exclusion. Then Aristotle’s thesis for exclusion (again, in two versions) can be formulated as follows:
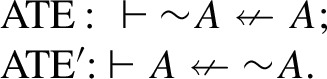


ATE basically says that every proposition excludes its own negation, and ATE$^\prime$ says that any proposition is excluded by its own negation. In terms of information, one can understand this as saying that the information that *A* is true provides the information that ${\sim }A$ is false, whereas ATE$^\prime$ says that the information that ${\sim }A$ is true provides the information that *A* is false. This does not exclude the simultaneous satisfiability of *A* and ${\sim }A$ in the sense that both *A* and ${\sim }A$ receive support of truth. Aristotle’s thesis for exclusion is dual to its original counterpart for implication. This can be justified by the *principle of duality* as it was formulated, e.g. by Church for classical logic, see (Church, 1956, pp. 106–108):

Let *A* be some formula of our language, and let $A^d$ be obtained by replacing in *A* each occurrence of the logical constants (in particular, propositional connectives) with their duals, leaving the self-dual operators (in particular, negation) as is. Then $\vdash \!A \Leftrightarrow \;\vdash \!{\sim }A^d$.

If we turn now to the co-implicative analogue of Boethius’ thesis, it appears that the situation there is not so straightforward. Generally, Boethius’ thesis for exclusion should establish a connection between two (maybe different) propositions, one of which (does not) exclude (the negation of) the other. It seems natural to assume that two statements, one saying that some proposition does not exclude (the negation of) another proposition, and the other saying that the first proposition does exclude (the negation of) the first proposition, are compatible in the sense that none of these statements excludes the other. This intuition can be expressed as follows:
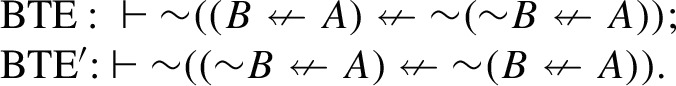


In a rule form the intuition underlying Boethius’ thesis for exclusion becomes even more transparent, saying that if some proposition does not exclude (the negation of) another proposition, then it follows that the first proposition does exclude (the negation of) that other proposition:
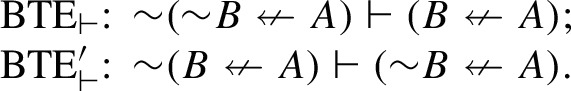


By way of example, let *A* stand for “it is raining”, and *B* mean “the road is wet”. Then, since it is surely not the case that rain excludes a wet road, we can derive, using BTE$^\prime _\vdash$, that rain excludes that the road is not wet. It should be noted that Boethius’ thesis for exclusion thus formulated, unlike Aristotle’s thesis, is not exactly dual to its implicational counterpart. In particular, the genuine dual of BT would be $\vdash {\sim }((A \nleftarrow B) \nleftarrow {\sim }(A \nleftarrow {\sim }B))$, and the dual of BT$_\vdash$ would be ${\sim }(A\nleftarrow {\sim }B)\vdash (A\nleftarrow B)$. However, this version of Boethius’ thesis for exclusion seems rather non-intuitive.

Thus, a logical system in a language containing $\nleftarrow$ and $\sim$ can be called a *logic of connexive exclusion* (or a connexive logic of exclusion) if and only if it obeys ATE, ATE$^\prime$, BTE, BTE$^\prime$, and, in addition, the connective of exclusion in it is asymmetric, thus being different from a simple mutual incompatibility of propositions. That is, $A \nleftarrow B \vdash B \nleftarrow A$ does not hold. If instead of BTE and BTE$^\prime$, we accept BTE$_\vdash$ and BTE$^\prime _\vdash$, then the corresponding logic will be called a *weakly* connexive logic of exclusion.

## A Semantics for Connexive Exclusion

Given a general duality between implication and exclusion, it is natural to suppose that connexive exclusion can be obtained from a connexive implication by a suitable procedure of dualisation. As already said, one can find in the literature quite a few systems of connexive logic dealing with different versions of connexive implication. Here we adopt as our basic logic of connexive implication the system $\textsf{C}$ from (Wansing, 2005), which according to Graham Priest (2008, p. 178), where C is called ‘*W*’, is “one of the simplest and most natural” connexive logics. The system $\textsf{C}$ is endowed with conditions of truth support for intuitionistic implication, but provides specific (connexive) conditions of falsity support.

System $\textsf{C}$ is formulated in the following language $\mathcal{L}^{C}$:

$$\begin{aligned} A {:}{:} = p \mid (A\wedge A) \mid (A \vee A) \mid (A \rightarrow A) \mid {\sim }A. \end{aligned}$$

The semantic characterization of $\textsf{C}$ just mentioned employs Kripke frames as defined above. A C-model based on a Kripke frame $\mathfrak {F}$ is a structure $\mathfrak {M}^{C} \,{=}\, \langle W, \le , v^{+}, v^{-}\rangle$, where $v^{+}$ and $v^{-}$ are valuation functions from the set of atomic formulas into $\mathcal {P}(W)$, subject to the conditions (FH$^+$) and (FH$^+$). Note that (*con*) and (*com*) are not required.

Relations $\models ^+$ and $\models ^-$ in C-models are defined by means of ($p_+$), ($p_-$), ($\wedge _+$), ($\wedge _-$), ($\vee _+$), ($\vee _-$) from intuitionistic and dual intuitionistic models, and the following conditions for $\sim$ and $\rightarrow$: 
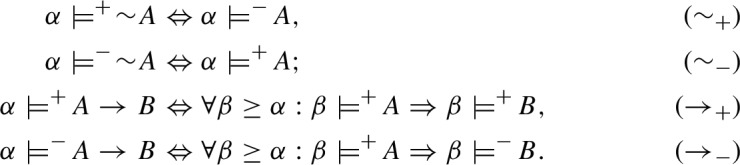


Validity and entailment for C-models are defined as in Definition 4 by replacing $\mathfrak {M}^{I}$ with $\mathfrak {M}^{C}$.

As one can see, in C-models, ($\rightarrow _+$) is the same as ($\succ _+$) from I-models, while ($\rightarrow _-$) differs from ($\succ _-$) in that it is not simply the meta-language (classical) negation of ($\rightarrow _+$), but expresses some general connection between antecedent and consequent. In this respect ($\rightarrow _-$) is on a par with ($\rightarrow _+$). Note that ($\rightarrow _-$) differs also from the falsity conditions of implication in Nelson’s logics of constructible falsity. In the latter, a state supports the falsity of $A \rightarrow B$ iff *this very state* supports the truth of *A* and falsity of *B*. In contrast, ($\rightarrow _-$) is dynamic, involving for the evaluation of $A\rightarrow B$ at some state not only this state itself, but also the set of all accessible states. In this way implication in $\textsf{C}$ becomes fully connexive both in its truth conditions and in its falsity conditions. AT, AT$^\prime$, BT, BT$^\prime$, BT$_\vdash$ and BT$^\prime _\vdash$ are all valid in $\textsf{C}$. Since the converses of BT and BT$^\prime$ are valid as well, C is also said to be *hyperconnexive*.^5^

Since (*con*) and (*com*) are not required in C-models, the latter allow under-determined and over-determined valuations. Thus, $\textsf{C}$ is paraconsistent and paracomplete, in the sense that neither ${\sim }L\models _{\textsf{C}}\!A$, nor $A\models _{\textsf{C}}\!L$ hold (where *L* is logically valid, and *A* is any formula). Moreover, $\textsf{C}$ is a contradictory logic even being non-trivial, for example, we have: $\models _{\textsf{C}} (p \wedge {\sim }p) \rightarrow ({\sim }p \vee p)$ and $\models _{\textsf{C}} {\sim }((p \wedge {\sim }p) \rightarrow ({\sim }p \vee p))$ as well as $\models _{\textsf{C}} p \rightarrow ({\sim }p \rightarrow p)$ and $\models _{\textsf{C}} {\sim }(p \rightarrow ({\sim }p \rightarrow p))$.

Note that there is also the system $\textsf{C}^\bot$ that expands the axiomatic proof system for $\textsf{C}$ by a falsity constant, $\bot$, governed by the axioms $\bot \rightarrow A$ and ${\sim }\bot$, cf. Fazio and Odintsov (2024).

Now, building on the connexive implication thus defined, we can obtain a rather natural semantic characterisation of the connexive exclusion. Just as the semantic definition of the connexive implication accepts the truth conditions of the intuitionistic implication and formulates specific (connexive) falsity conditions, so the semantic definition of the connexive exclusion, in a dual way, accepts the falsity conditions of the dual intuitionistic implication and formulates specific (connexive) truth conditions.

Consider the language $\mathcal{L}^{CE}$ for the logic of connexive exclusion $\textsf{CE}$, obtained from $\mathcal{L}^{C}$ by replacing the connective of implication ($\rightarrow$) with the exclusion ($\nleftarrow$). CE-models based on a Kripke frame $\mathfrak {F}$ are obtainable from C-models simply by taking the following definitions of $\models ^+$ and $\models ^-$ for $\nleftarrow$ instead of those for $\rightarrow$: 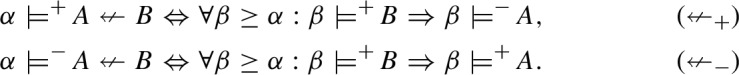


Dually to the conditions for $\rightarrow$ in C-models, ($\nleftarrow _-$) in CE-models is the same as ($\nprec _-$) from DI-models, whereas ($\nleftarrow _+$) presents a kind of a connexive generalization of ($\nprec _+$). It is also noteworthy that the truth conditions of $A\nleftarrow B$ coincide with the falsity conditions of $B\rightarrow A$ and the falsity conditions of $A \nleftarrow B$ are the same as the truth conditions of $B\rightarrow A$. This correlation between the truth and falsity conditions of implication and dual implication is characteristic and exactly the same as in classical logic, as well as in intuitionistic and dual intuitionistic logics.

Incidentally, one might expect that in moving from C-models to CE-models we would have to replace the heredity conditions FH$^+$ and FH$^-$ with their “dual counterparts” BH$^+$ and BH$^-$ (just as by the transition from I-models to DI-models we switched from FH$^+$ and BH$^-$ to BH$^+$ and FH$^-$). However, retaining FH$^+$ and FH$^-$ also in CE-models is well-justified. Indeed, recall that the semantic definition of a connexive exclusion adopts the condition of falsity support of the dual intuitionistic implication, and the latter is subject to FH$^-$. On the other hand, the condition of truth support for $\nleftarrow$ (unlike for $\nprec$) presents a *general statement*, and as such must also be preserved forwards.

Validity and entailment for CE-models are again defined as in Definition 4 by replacing $\mathfrak {M}^{I}$ with $\mathfrak {M}^{CE}$.

### Observation 1

ATE, ATE$^\prime$, BTE, BTE$^\prime$, BTE$_\vdash$, and BTE$^\prime _\vdash$ are valid in every CE-model.

### Proof

Consider ATE. Assume, there is $\mathfrak {M}^{CE}$, there is ${\alpha }\in W$, such that $\not \models ^+\!{\sim }A \nleftarrow A$. By ($\nleftarrow _+$), $\exists {\beta }\ge {\alpha }: {\beta }\models ^+\!A \text{ and } {\beta }\not \models ^-\!{\sim }A$. By ($\sim _-$), ${\beta }\models ^+\!A \text{ and } {\beta }\not \models ^+\!A$, a contradiction. ATE$^\prime$ is checked analogously. Next, consider BTE$^\prime$. Assume, there is $\mathfrak {M}^{CE}$, there is ${\alpha }\in W$, such that ${\alpha }\not \models ^+{\sim }(({\sim }B \nleftarrow A) \nleftarrow {\sim }(B \nleftarrow A))$. Then, by ($\sim _+$), ${\alpha }\not \models ^-(({\sim }B \nleftarrow A) \nleftarrow {\sim }(B \nleftarrow A))$. By ($\nleftarrow _+$), $\exists {\beta }\ge {\alpha }: {\beta }\models ^+{\sim }(B \nleftarrow A) \text{ and } {\beta }\not \models ^+ ({\sim }B \nleftarrow A)$. Hence, by ($\sim _+$), ($\nleftarrow _-$), ($\nleftarrow _+$), and ($\sim _-$) we obtain $\forall {\gamma }\ge {\beta }: {\gamma }\models ^+A \Rightarrow {\gamma }\models ^+B$, and $\exists {\gamma }\ge {\beta }: {\gamma }\models ^+ A \text{ and } {\gamma }\not \models ^+B$. A contradiction. BTE is checked similarly. Now, let us check BTE$_\vdash$. Assume, there is $\mathfrak {M}^{CE}$, there is ${\alpha }\in W$, such that ${\alpha }\models ^+\!{\sim }(B\nleftarrow A)$ and ${\alpha }\not \models ^+\!({\sim }B\nleftarrow A)$. By ($\sim _+$), we first have ${\alpha }\models ^-\!(B\nleftarrow A)$ and ${\alpha }\not \models ^+\!({\sim }B\nleftarrow A)$. Then, by ($\nleftarrow _-$), ($\nleftarrow _+$) and ($\sim _+$), we get $\forall \beta \ge \alpha : {\beta }\models ^+\!A \Rightarrow {\beta }\models ^+\! B$ and $\exists {\beta }\ge \alpha : {\beta }\models ^+\!A \text{ and } {\beta }\not \models ^+\! B$, whence we easily obtain a contradiction. BTE$^\prime _\vdash$ is checked in the same way. $\square$

Also, $\nleftarrow$ is non-commutative, since it is not difficult to construct a CE-model $\mathfrak {M}^{CE}$, in which there is ${\alpha }\in W$, such that ${\alpha }\models ^+ A\nleftarrow B$, but ${\alpha }\not \models ^+ B\nleftarrow A$ for some *A* and *B*. Thus, CE-models defined as above indeed determine a logic of connexive exclusion (both strong and weak) as defined at the end of the previous section. A remarkable feature of this logic is that it is contradictory, just like $\textsf{C}$, in the sense that there are valid formulas whose negations are also valid. Among these formulas are, for example, $\models _{\textsf{CE}}({\sim }p \vee p) \nleftarrow (p \wedge {\sim }p)$ and $\models _{\textsf{CE}} {\sim }((p \vee {\sim }p) \nleftarrow ({\sim }p \wedge p))$. However, since $\textsf{CE}$ is paraconsistent, it is non-trivial, even being negation inconsistent.

Our next task will be to formalize this logic deductively by a suitable proof system.

## A Binary Consequence System

CE-models constructed in the previous section are not bivalent. Since neither (*con*) nor (*com*) is accepted, the following *four* combinations of assignments of the valuation functions $v^+$ and $v^-$ to *p* for any state ${\alpha }\in W$ are possible:

- (1) $${\alpha }\in v^+(p)$$ and $${\alpha }\notin v^-(p)$$,       (2) $${\alpha }\not \in v^+(p)$$ and $${\alpha }\in v^-(p)$$,
- (3) $${\alpha }\notin v^+(p)$$ and $${\alpha }\notin v^-(p)$$,       (4) $${\alpha }\in v^+(p)$$ and $${\alpha }\in v^-(p)$$;

and the same holds true for the relations $\models ^+$ and $\models ^-$ with respect to any formula *A*. That is, in addition to the two classical assignments (1) and (2), we have two non-classical assignments (3) and (4), namely the underdetermined and overdetermined ones. In this sense the proposed semantic construction for $\textsf{CE}$ belongs to the paradigm of a four-valued logic developed and justified by Dunn and Belnap, see, e.g. (Omori & Wansing, 2019). Therefore, to begin with, it seems quite natural to explicate the logic of connexive exclusion determined by CE-models in the context of Dunn and Belnap’s four-valued approach.

The four-valued Belnap-Dunn logic is standardly implemented as the logic of *first-degree entailment* ($\textsf{FDE}$). There are quite a few different versions of $\textsf{FDE}$, realized by means of various deductive systems, see, e.g., (Omori & Wansing, 2017). One of such realizations, and historically the first, is formulated as a so-called *binary consequence system*, see (Dunn, 1999). A binary consequence system is a proof system, which manipulates *binary consequence expressions* (or simply *binary consequences*). More formally, see (Shramko, 2022, p. 376):

### Definition 5

Let *A* and *B* be some single formulas of our language. Then a binary consequence expression (or simply a consequence) is an expression of the form $A \vdash B$, where $\vdash$ is a consequence relation. A binary consequence rule is a construction of the form

$$\begin{aligned} \displaystyle {\frac{\mathcal{C}_1, \ldots , \mathcal{C}_n}{\mathcal{C}}}, \end{aligned}$$

where $\mathcal{C}_1, \ldots , \mathcal{C}_n, \mathcal{C}$ are binary consequence expressions. If there are no $\mathcal{C}_1, \ldots , \mathcal{C}_n$ (i.e., $n= 0$), the rule is an axiom scheme. A binary consequence rule is proper iff $n \ge 1$. A binary consequence system is a nonempty set of binary consequence rules, of which at least one is an axiom. A binary consequence system is proper iff it has at least one proper binary consequence rule.

Thus, binary consequence systems belong to the Fmla-Fmla logical framework in the sense of (Humberstone, 2011, p. 108), dealing with consequence relations between *single formulas*. A binary consequence expression, which belongs to this framework, can be viewed as a particular (restricted) case of a sequent in Gentzen’s sense, where antecedent and succedent are both singletons. The logic of first-degree entailment was formalized in (Anderson & Belnap, 1975, § 15.2) exactly as a binary consequence system. Namely, consider the language $\mathcal{L}^{FDE}$:

$$\begin{aligned} A {:}{:} = p \mid (A\wedge A) \mid (A \vee A) \mid {\sim }A. \end{aligned}$$

System $\textsf{FDE}$ is determined by the following axioms and rules of inference:
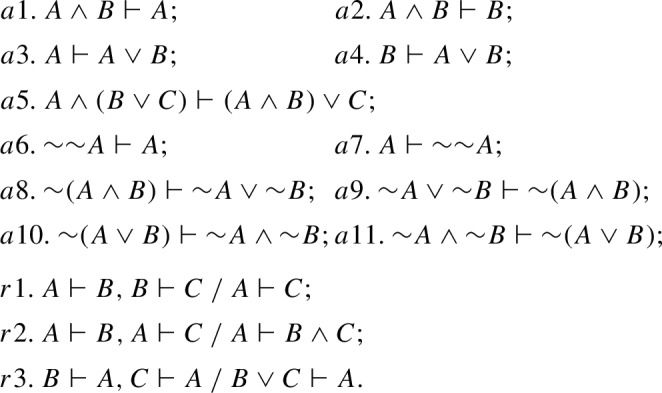


To obtain the *binary consequence system for connexive exclusion* $\mathsf {CE_c}$ we switch to the language $\mathcal{L}^{CE}$ and expand $\textsf{FDE}$ with the following axioms and rule of inference that govern the connective of connexive exclusion $\nleftarrow$: 
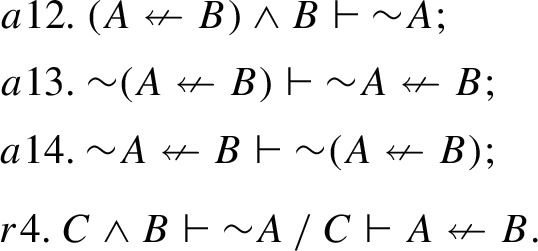


Here, *a*13 is exactly BTE$^\prime _\vdash$, and *a*14 is its converse. BTE$_\vdash$ is easily derivable in $\mathsf {CE_c}$ by using *a*12, *a*13 and *r*4. As for the “exclusion version” of Aristotle’s thesis and strong Boethius’ thesis, they turn out to be inexpressible in $\mathsf {CE_c}$, since all $\mathsf {CE_c}$-statements are binary consequence expressions, whereas Aristotle’s thesis, as well as strong Boethius’ thesis (both for implication and exclusion) have the form of a theorem. Instead, one can derive in $\mathsf {CE_c}$ a statement that can be called *Aristotle’s restricted thesis for exclusion*, which is a particular case of Boethius’ rule for exclusion. Two versions of this thesis in a rule form can be formulated as follows:
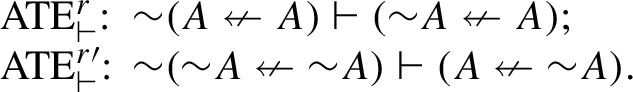


Taking into account that ${\sim }(A\nleftarrow A)$ is the dual of the identity law ($A\rightarrow A$), and is thus valid in any CE-model, the antecedent of the consequence expressions ATE$^r_\vdash$ and ATE$^{r\prime }_\vdash$ could be detached, if consequence expressions with empty antecedents were allowed, and in this way one could obtain Aristotle’s thesis for exclusion in its unrestricted form.

Incidentally, the contradictions, which, as noted above, happen to be valid in the class of CE-models at the level of semantic tautologies, are also inexpressible in the system $\mathsf {CE_c}$. This “elimination of contradictoriness” comes here at the expense of restricting the deductive apparatus to the Fmla-Fmla framework, and thus the mentioned contradictions are simply left out of consideration within this limited framework. Clearly, in systems without such a limitation, the phenomenon of logical negation inconsistency characteristic of CE-models will again be fully manifested.

The fact that $A\vdash B$ is derivable in $\mathsf {CE_c}$ will be denoted by $A \vdash _{\mathsf {CE_c}}\!\!B$. Checking that $\mathsf {CE_c}$ is sound with respect to CE-models is routine. Completeness can be established by a canonical model construction. Let, as usual, a CE-theory *t* be a set of formulas of the language $\mathcal{L}^{CE}$ closed under consequences provable in ${\mathsf {CE_c}}$ and conjunction. That is, (1) if $A\in t$ and $A \vdash _{\mathsf {CE_c}}\!\!B$, then $B\in t$; and (2) if $A\in t$ and $B\in t$, then $A\wedge B\in t$. A CE-theory *t* is *prime* iff it has the disjunction property (if $A \vee B \in t$, then $A \in t$ or $B \in t$), and it is *trivial* iff it contains all the formulas of $\mathcal{L}^{CE}$. The following Lindenbaum lemma is well known, and we formulate it here with respect to CE-theories:

### Lemma 1

For any *A* and *B*:

1. If $A \not \vdash _{\mathsf {CE_c}}\!\!B$, then there exists a prime CE-theory *t* such that $A \in t$ and $B \notin t;$
2. If *t* is a CE-theory, such that $A \notin t$, then *t* can be extended to a prime CE-theory $t^\prime$ such that $A \notin t^\prime$.

### Proof

1. Suppose $A \not \vdash _{\mathsf {CE_c}}\!\!B$. Consider a CE-theory $t_0 = \{C: A \vdash _{\mathsf {CE_c}}\!\!C\}$. Clearly, $A \in t_0$ and $B\notin t_0$. Let $t + A$ means the smallest CE-theory $t^\prime$, such that $t \cup \{A\} \subseteq t^\prime$. Now we enumerate all the formulas of $\mathcal{L}^{CE}$ $A_1, A_2, \ldots$, and construct a series of CE-theories $t_1, t_2, \ldots$, where for each *n*, $t_{n+1} = t_n + A_{n+1}$ if $t_n + A_{n+1} \not \vdash _{\mathsf {CE_c}}\!\!B$, otherwise $t_{n+1} = t_n$. The required CE-theory *t* is obtained then as the union of all the $t_n$’s from the series of CE-theories so constructed. It is easy to see that $A \in t$, and *t* is the maximal CE-theory, such that $B \notin t$. To see that *t* is prime, suppose that $D\notin t$ and $D^\prime \notin t$, whereas $D \vee D^\prime \in t$. Consider the theories $t + D$ and $t + D^\prime$. Due to maximality of *t*, we have $B\in t + D$, and also $B\in t + D^\prime$. This means that there are some formulas $E_1,\ldots ,E_n \in t$, such that $E_1 \wedge \ldots \wedge E_n \wedge D \vdash _{\mathsf {CE_c}}\!\!B$, and $E_1 \wedge \ldots \wedge E_n \wedge D^\prime \vdash _{\mathsf {CE_c}}\!\!B$. By *r*3 we get $(E_1 \wedge \ldots \wedge E_n \wedge D) \vee (E_1 \wedge \ldots \wedge E_n \wedge D^\prime )\vdash _{\mathsf {CE_c}}\!\!B$, and from this, using *a*5, we can obtain $(E_1 \wedge \ldots \wedge E_n) \wedge (D \vee D^\prime )\vdash _{\mathsf {CE_c}}\!\!B$. But this would mean that $B \in t$, a contradiction.
2. Let *t* be a CE-theory, such that $A \notin t$. Then for any formula $B\in t$ we have $B\not \vdash _{\mathsf {CE_c}}\!\!A$. The required prime CE-theory $t^\prime$ can be constructed by the procedure described above. $\square$

In what follows 1.1 will be directly used in the proof of the completeness Theorem 1, whereas 1.2 is needed to extend the canonical valuation to the connective of connexive exclusion (Lemma 2).

Next, we formulate the canonical model lemma as follows:

### Lemma 2

Define the canonical CE-model $$\mathfrak {M}^{CE}_c = \langle W^{CE}_c, \le _c, v^+_c, v^-_c\rangle $$, so that $$ W^{CE}_c$$ is the set of all non-trivial prime CE-theories, $$\le _c$$ is the subset relation on $$W^{CE}_c$$, and $$v^+_c$$ and $$v^-_c$$ are defined for any propositional variable *p* and any $$t\in W_c$$ as follows: $$t\in v^+_c(p) \Leftrightarrow p\in t$$, and $$t\in v^-_c(p) \Leftrightarrow {\sim }p\in t$$. Then $$\mathfrak {M}^{CE}_c$$ is indeed a CE-model, and in particular, for any $$t\in W^{CE}_c$$, and any formula *A*: $$t\models ^+_c \!A \Leftrightarrow A\in t$$, and $$t\models ^-_c\!A\Leftrightarrow {\sim }A\in t$$.

### Proof

First of all, we note that (FH$^+$) and (FH$^-$) obviously hold for the canonical CE-model. An easy induction extends these conditions for any formula *A*. Now, one has to show that $v^+_c$ and $v^+_c$ determine the corresponding relations $\models ^+_c$ and $\models ^-_c$, so that for any $t\in W^{CE}_c$, and for any *A*: $t\models ^+_c \!A \Leftrightarrow A\in t$ and $t\models ^-_c\!A\Leftrightarrow {\sim }A\in t$.

For propositional variables, this holds directly by definitions of $v^+_c$ and $v^+_c$. For other formulas it can be proved by course-of-values induction on the number of (occurrences of) propositional connectives in *A*. We show here only the induction step for $\nleftarrow$ (the cases for $\wedge$, $\vee$ and $\sim$ are well known).

First, we have to show that $A\nleftarrow B \in t \Leftrightarrow \forall t^\prime \supseteq t: B \in t^\prime \Rightarrow {\sim }A \in t^\prime$. From left to right, let $A\nleftarrow B \in t$, and consider an arbitrary $t^\prime \supseteq t$, such that $B \in t^\prime$. We have $A\nleftarrow B \in t^\prime$, and hence, $B\wedge (A\nleftarrow B) \in t^\prime$. By *a*12, we get ${\sim }A \in t^\prime$. For the converse, assume $A\nleftarrow B \notin t$. Consider theory $t+B$. Observe that ${\sim }A\notin t+B$. Otherwise, there would be some finite set {$C_1, \ldots , C_n\} \subseteq t$, such that $(C_1 \wedge \ldots \wedge C_n) \wedge B\vdash _{\mathsf {CE_c}}\!{\sim }A$. By *r*4, we then would get $C_1 \wedge \ldots \wedge C_n \vdash _{\mathsf {CE_c}}\!A\nleftarrow B$, from which it would follow that $A\nleftarrow B \in t$, a contradiction. By Lemma 1.2, there is a prime CE-theory $t^\prime \supseteq t + B$, such that ${\sim }A\notin t^\prime$.

It remains to show that ${\sim }(A\nleftarrow B) \in t \Leftrightarrow \forall t^\prime \supseteq t: B \in t^\prime \Rightarrow A \in t^\prime$. From left to right, let ${\sim }(A\nleftarrow B) \in t$. By *a*13, we get ${\sim }A\nleftarrow B \in t$. Consider arbitrary $t^\prime \supseteq t$, such that $B\in t^\prime$. Obviously, ${\sim }A\nleftarrow B \in t^\prime$, and hence, $B \wedge ({\sim }A\nleftarrow B) \in t^\prime$. By *a*12, we obtain ${\sim \sim }A\in t^\prime$, and finally, by *a*6, $A\in t^\prime$. If we now assume that ${\sim }(A\nleftarrow B) \notin t$, we again may wish to consider the theory $t + B$. Then, $A\notin t + B$, since otherwise, there would be some finite set {$C_1, \ldots , C_n\} \subseteq t$, such that $(C_1 \wedge \ldots \wedge C_n) \wedge B\vdash _{\mathsf {CE_c}}\!A$; by *a*7, we obtain $(C_1 \wedge \ldots \wedge C_n) \wedge B\vdash _{\mathsf {CE_c}}\!{\sim \sim }A$; and by *r*4, we then would get $C_1 \wedge \ldots \wedge C_n \vdash _{\mathsf {CE_c}}\!{\sim }A\nleftarrow B$; finally, using *a*14, we get $C_1 \wedge \ldots \wedge C_n \vdash _{\mathsf {CE_c}}\!{\sim }(A\nleftarrow B)$, and hence, ${\sim }(A\nleftarrow B)\in t$, a contradiction. By Lemma 1.2, there is a prime CE-theory $t^\prime \supseteq t + B$, such that $A\notin t^\prime$. $\square$

Now, the completeness can be proved in the standard way.

### Theorem 1

$$A \models _{\mathsf {CE_c}}\!B \Rightarrow A \vdash _{\mathsf {CE_c}}\!B$$.

### Proof

As usual, assume, for contraposition, that $A \not \vdash _{\mathsf {CE_c}}\!B$. By Lemma 1.1, there exists a (non-trivial) prime CE-theory *t* such that $A \in t$ and $B \notin t$. Consider the canonical model $\mathfrak {M}^{CE}_c$. By Lemma 2, we have $t\models ^+\!A$ and $t\not \models ^+ \!B$. Hence, $A \not \models _{\!{{ {\mathfrak {M}^{\!{{{CE}}}}_c}}}}\!B$, and thus, $A \not \models _{\mathsf {CE_c}}\!B$. $\square$

Thus, $\mathsf {CE_c}$ accurately represents, at the level of logical deduction, the binary consequence relation between formulas of the language of connexive exclusion $\mathcal{L}^{CE}$. Moreover, the system $\textsf{CE}_c$ has the weak replacement property. Let *C*(*A*) stand for the result of uniformly replacing all occurrences of a certain propositional variable *p* in *C* by *A*. Then *C*(*B*) is the result of replacing all occurrences of the same propositional variable *p* in *C* by *B*. Usually, the mutual derivability of formulas *A* and *B* results in a replacement property; the formulas can be replaced by each other without affecting the mutual derivability of the formulas in which the substitution is performed. In logics with strong negation, such as David Nelson’s constructive logics and the connexive logic C, the requirements for replaceability often are higher. Note that also in ${\mathsf {CE_c}}$ mutual derivability is not a congruence relation, i.e., an equivalence relation satisfying the replacement property, cf., e.g., (Dunn & Hardegree, 2001, p. 21), which means that the replacement ruledoes not preserve validity, where $A \dashv \vdash _{\mathsf {CE_c}} B$ is an abbreviation of $A\vdash _{\mathsf {CE_c}} B$ and $B\vdash _{\mathsf {CE_c} } A$. We have ${\sim }(p \nleftarrow p)$ ${({\sim }p\nleftarrow p)\dashv \vdash _{\mathsf {CE_c}}{\sim }(q\nleftarrow q)}$, but we do not have ${{\sim }{\sim }(p\nleftarrow p) {\dashv \vdash _{\mathsf {CE_c}}}}$ ${{\sim }{\sim }(q \nleftarrow q)}$. However, the rule of weak replacement, i.e.,is validity preserving. This follows from Theorems 1 and 6 together with Theorem 7 in Sect. 5.

## A Sequent Calculus

As already pointed out, ${\mathsf {CE_c}}$, being a binary consequence system, is rather restrictive. In this system we can derive Boethius’ thesis for exclusion in a rule form and what we have called “restricted Aristotle’s thesis for exclusion”, also in the form of a rule. But Aristotle’s and Boethius’ theses for exclusion in their entirety are not expressible within the framework of ${\mathsf {CE_c}}$. Therefore, we will proceed to the construction of an (unrestricted) sequent calculus ${\textsf{LCE}}$, in which ATE, ATE$^\prime$, BTE, and BTE$^\prime$ will be derivable. Then it will be shown that ${\textsf{LCE}}$ is complete with respect to CE-models. As $\textsf{CE}$ is definitionally equivalent with $\textsf{C}$, the results of this section are straightforwardly obtained from known similar results about $\textsf{C}$.

We use $\Gamma$, $\Delta$, $\Sigma$ as variables for finite sets of formulas. A sequent is an expression of the form $\Delta \Rightarrow A$, and $\Delta$ is said to be the antecedent of $\Delta \Rightarrow A$ and *A* its succedent. We write $A \Rightarrow B$ instead of $\{A\} \Rightarrow B$ and $\Delta , \Gamma \Rightarrow A$ ($A, \Gamma \Rightarrow B$) instead of $\Delta \cup \Gamma \Rightarrow A$ ($\{A\} \cup \Gamma \Rightarrow B$). A sequent calculus is a non-empty set containing some axiomatic sequent rules, also called ‘initial sequents’, and rules of the formwhere *seq* and all $seq_i$ $(1\le i\le n)$ are sequents. Derivations in a sequent calculus are inductively defined. Every instance of an axiomatic sequent is a derivation, and applications of sequent rules to instances of their schematic premise sequents as conclusions of derivations result in a derivation. If there is a derivation of a sequent *seq* in a sequent calculus $\textit{Calc}$, we say that *seq* is provable in $\textit{Calc}$ and write $\textit{Calc} \vdash seq$. A rule of inference *R* is *admissible* in a sequent calculus $\textit{Calc}$ if for any instance

### Definition 6

The initial sequents of ${\textsf{LCE}}$ are of the form

$$\begin{aligned} { p \Rightarrow p } \quad \quad \quad { {\sim }p \Rightarrow {\sim }p } \end{aligned}$$

for any propositional variable *p*.

The structural inference rules of ${\textsf{LCE}}$ are of the form:The logical inference rules of ${\textsf{LCE}}$ are of the form:

### Remark 2

Note that working with finite sets as antecedents of sequents means that there may be hidden contractions of occurrences of assumptions in derivations. The last step in the following derivation, for example, is an application of $(\nleftarrow {\textrm{r}})$:

By induction on the complexity of *A*, one can show that for any $\mathcal {L}^{CE}$-formula *A*, $\textsf{LCE}$ $\vdash A \Rightarrow A$. The axioms and rules of $\textsf{CE}_{\textsf{c}}$ can easily be derived in $\textsf{LCE}$ when ‘$\vdash$’ is replaced in $\textsf{CE}_{\textsf{c}}$ with ‘$\Rightarrow$’. For the axioms and the rule that involve $\nleftarrow$, i.e., $a13-a14$ and rule *r*4, we have the following derivations:Moreover, we have the following simple derivations of ATE, ATE$^\prime$, BTE, and BTE$^\prime$ in $\textsf{LCE}$:For two of the earlier example of a valid contradiction in CE-models, i.e., a formula *A* such that both $\models _{\textsf{CE}} A$ and $\models _{\textsf{CE}} {\sim }A$, we have:Let $\mathcal {L}^+$ be the language of positive, negation-free intuitionistic logic, $\mathsf {Int^+}$, with the two-place connectives $\wedge$, $\vee$, and $\succ$, defined over a denumerable set $\textsf{Prop}$ of propositional variables. As in the case of $\textsf{C}$, completeness can be shown by means of a faithful embedding into $\mathsf {Int^+}$, cf. (Wansing, 2005). For that purpose, we present the sequent calculus $\mathsf {LInt^+}$ for $\mathsf {Int^+}$.

### Definition 7

The initial sequents of $\mathsf {LInt^+}$ are of the form ${ p \Rightarrow p }$, for any *p* $\in$ $\textsf{Prop}$. The structural inference rules of $\mathsf {LInt^+}$ are (cut) and (we). The logical inference rules of $\mathsf {LInt^+}$ are ($\wedge$l), ($\wedge$r), ($\vee$r1), ($\vee$r2), and

### Definition 8

Let $\textsf{Prop}'$ $=$ $\{p' \mid p \in \textsf{Prop} \}$. We inductively define the translation $\tau$ from $\mathcal {L}^{CE}$ into $\mathcal {L}^+$ based on $\textsf{Prop} \cup \textsf{Prop}'$ as follows (some outermost brackets are omitted):

$$\begin{aligned} \begin{array}{rclcrcl} \tau (p)& := & p & & \tau ({\sim }p) & := & p' \\ \tau (A \wedge B) & := & \tau (A ) \wedge \tau (B) & & \tau ({\sim }(A\wedge B)) & := & \tau ({\sim }A ) \vee \tau ({\sim }B) \\ \tau (A \vee B) & := & \tau (A ) \vee \tau (B) & & \tau ({\sim }(A\vee B)) & := & \tau ({\sim }A ) \wedge \tau ({\sim }B) \\ \tau (A \nleftarrow B) & := & \tau (B) \succ \tau ({\sim }A) & & \tau ({\sim }(A \nleftarrow B)) & := & \tau (B) \succ \tau (A) \\ & & & & \tau ({\sim }{\sim }A) & := & \tau (A). \end{array} \end{aligned}$$

We write $\tau (\Gamma )$ for $\{\tau (A) \mid A \in \Gamma \}$.

We first consider a syntactical embedding of $\textsf{LCE}$ into $\mathsf {LInt^+}$.

### Theorem 2

(Syntactical embedding) Let ${\Gamma }\cup \{A\}$ be a finite set of formulas of $\mathcal{L}^{CE}$ and $\tau$ be the translation defined in Definition 8. Then

1. $$\textsf{LCE} \vdash { {\Gamma } \Rightarrow A }$$ iff  $$\mathsf {LInt^+}$$ $$\vdash { \tau ({\Gamma }) \Rightarrow \tau (A) }$$,
2. $$\textsf{LCE}-\{\mathrm{(cut)}\} \vdash { {\Gamma } \Rightarrow A }$$ iff  $$\mathsf {LInt^+}\!-\!\{\mathrm{(cut)}\}$$ $$\vdash { \tau ({\Gamma }) \Rightarrow \tau (A) }$$.

### Proof

It is enough to consider (1) because claim (2) can be obtained from the proof of (1). For the direction from left to right we use induction on the construction of derivations in $\textsf{LCE}$. We present one case of the rules for $\nleftarrow$.

Case ($\nleftarrow {\textrm{l}}$): By the induction hypothesis, we have $\mathsf {LInt^+}$ $\vdash$ ${ \tau ({\Gamma }) \Rightarrow \tau (B) }$ and $\mathsf {LInt^+}$ $\vdash$ ${ \tau ({\sim }A), \tau (\Delta ) \Rightarrow \tau (C) }$. We then obtainin $\mathsf {LInt^+}$, where $\tau (B) \succ \tau ({\sim }A)$ = $\tau (A \nleftarrow B)$.

For the direction from right to left we use induction on the construction of derivations in $\mathsf {LInt^+}$. For the case of (cut) we haveBy induction on the construction of *A*, we can show that for *A* in $\mathcal{L}^+$ defined over $\textsf{Prop}$, $A = \tau (A)$. Then, by the induction hypothesis, we have $\textsf{LCE}$ $\vdash$ ${ {\Gamma } \Rightarrow A }$ and $\textsf{LCE}$ $\vdash$ ${ A, \Sigma \Rightarrow C }$. We obtain $\textsf{LCE}$ $\vdash$ ${ {\Gamma }, \Sigma \Rightarrow C }$ by using (cut) in $\textsf{LCE}$. $\square$

### Theorem 3

(Cut-admissibility) The rule (cut) is admissible in cut-free $\textsf{LCE}$.

### Proof

Suppose $\textsf{LCE}$ $\vdash$ ${ {\Gamma } \Rightarrow A }$. Then, by Theorem 2 (1), $\mathsf {LInt^+}$ $\vdash$ ${ \tau ({\Gamma }) \Rightarrow \tau (A) }$, and $\mathsf {LInt^+} \!-\!\{\mathrm{(cut)}\}$ $\vdash$ ${ \tau ({\Gamma }) \Rightarrow \tau (A) }$ by the well-known cut-admissibility theorem for intuitionistic logic and thereby also $\mathsf {LInt^+}$, see, e.g., (Borisavljević, 1999). By Theorem 2 (2), we obtain $\textsf{LCE}-$(cut) $\vdash$ ${ {\Gamma } \Rightarrow A }$. $\square$

The cut-admissibility result allows one to note some important properties of $\textsf{LCE}$.

### Corollary 1

$\textsf{LCE}$ is paraconsistent with respect to ${\sim }$ and $\wedge$, and paracomplete with respect to ${\sim }$ and $\vee$ in the following sense: $\textsf{LCE}$ $\not \vdash A \wedge {\sim }A \Rightarrow B$ and $\textsf{LCE}$ $\not \vdash B \Rightarrow A \vee {\sim }A$.

### Corollary 2

For any $\mathcal {L}^{CE}$-formulas *A*, *B*, the following holds.

(Disjunction property) $\textsf{LCE}$ $\vdash \varnothing \Rightarrow A\vee B$ iff ($\textsf{LCE}$ $\vdash \varnothing \Rightarrow A$ or $\textsf{LCE}$ $\vdash \varnothing \Rightarrow B$).

(Constructible falsity) $\textsf{LCE}$ $\vdash \varnothing \Rightarrow {\sim }(A\wedge B)$ iff ($\textsf{LCE}$ $\vdash \varnothing \Rightarrow {\sim }A$ or $\textsf{LCE}$ $\vdash \varnothing \Rightarrow {\sim }B$).

### Theorem 4

(Decidability) $\textsf{LCE}$ is decidable.

### Proof

By the decidability of $\mathsf {Int^+}$, for each $\mathcal{L}^{\textsf{CE}}$-formula *A*, it is possible to decide whether $\tau (A)$ is provable in a proof system for $\mathsf {Int^+}$. Hence, by Theorem 2, $\textsf{LCE}$ is decidable. $\square$

The translation $\tau$ from Definition 8 also gives rise to a semantical embedding that can be used to obtain a completeness result for $\textsf{LCE}$. The entailment relation $\Gamma \models _{\mathsf {Int^+}} A$ between sets $\Gamma$ of $\mathcal{L}^+$-formulas and single $\mathcal{L}^+$-formulas *A* is defined as for the relation $\Gamma \models _{\textsf{CE}} A$ and the language $\mathcal{L}^{CE}$. It is well-known that $\mathsf {LInt^+}$ is sound and complete with respect to the class of all I-models $\langle W,\le , v\rangle$ (see Remark 1), i.e., $\mathsf {LInt^+}$ $\vdash \Gamma \Rightarrow A$ iff $\Gamma \models _{\mathsf {Int^+}} A$.

### Lemma 3

Let $\tau$ be the above mapping from $\mathcal {L}^{CE}$ into $\mathcal {L}^+$ based on $\textsf{Prop} \cup \textsf{Prop}'$, let $\mathfrak {M}^{\,\prime } =\langle W,\le ,v^+, v^-\rangle$ be a CE-model, and let $\mathfrak {M}$ = $\langle W,\le , v\rangle$, where the function *v* from $\textsf{Prop} \cup \textsf{Prop}'$ to subsets of *W* is defined by requiring for every $\alpha \in W$ and $p\in \textsf{Prop}$:

- $$\alpha \in v(p)$$ iff $$\alpha \in v^+(p)$$;
- $$\alpha \in v(p')$$ iff $$\alpha \in v^-(p)$$.

Clearly, $\mathfrak {M}$ is an I-model. For every $\mathcal {L}^{CE}$-formula *A* and $\alpha \in W$,

1. $${\mathfrak M}^{\,\prime }, \alpha \models ^+ A \text{ iff } \,{\mathfrak M}, \alpha \models \tau (A)$$,
2. $${\mathfrak M}^{\,\prime }, \alpha \models ^- A \text{ iff } \,{\mathfrak M}, \alpha \models \tau ({\sim }A)$$.

### Proof

By simultaneous induction on the construction of *A*. The set $\textsf{Prop}'$ of new propositional variable is used to deal with the case of strongly negated propositional variables ${\sim }p$. We present the case of formulas $B \nleftarrow C$.

$$\begin{aligned} \begin{array}{rll} & \mathfrak {M}^{\,\prime }, \alpha \models ^+ B \nleftarrow C & \\ \text{ iff } & \forall \beta \ge \alpha : \mathfrak {M}^{\,\prime }, \beta \models ^+ C \Rightarrow \mathfrak {M}^{\,\prime }, \beta \models ^- B & \\ \text{ iff } & \forall \beta \ge \alpha : \mathfrak {M}, \beta \models \tau (C) \Rightarrow \mathfrak {M}, \beta \models \tau ({\sim }B) & \text{ by } \text{ the } \text{ I.H. } \text{ for } \text{1. } \text{ and } \text{2. }\\ \text{ iff } & \mathfrak {M}, \alpha \models \tau (C) \succ \tau ({\sim }B) & \\ \text{ iff } & \mathfrak {M}, \alpha \models \tau (B \nleftarrow C) & \text{ by } \text{ the } \text{ definition } \text{ of } \tau . \end{array} \end{aligned}$$

$$\begin{aligned} \hspace{-6.5mm}\begin{array}{rll} & \mathfrak {M}^{\,\prime }, \alpha \models ^- B \nleftarrow C & \\ \text{ iff } & \forall \beta \ge \alpha : \mathfrak {M}^{\,\prime }, \beta \models ^+ C \Rightarrow \mathfrak {M}^{\,\prime }, \beta \models ^+ B & \\ \text{ iff } & \forall \beta \ge \alpha : \mathfrak {M}, \beta \models \tau (C) \Rightarrow \mathfrak {M}, \beta \models \tau (B) & \text{ by } \text{ the } \text{ I.H. } \text{ for } \text{1. }\\ \text{ iff } & \mathfrak {M}, \alpha \models \tau (C) \succ \tau (B) & \\ \text{ iff } & \mathfrak {M}, \alpha \models \tau ({\sim }(B \nleftarrow C)) & \text{ by } \text{ the } \text{ definition } \text{ of } \tau . \end{array} \end{aligned}$$

$$\square $$

### Lemma 4

Let $\tau$ be the above mapping from $\mathcal {L}^{CE}$ into $\mathcal {L}^+$ based on $\textsf{Prop}$ $\cup$ $\textsf{Prop}'$, let $\mathcal {M} =\langle W,\le ,v \rangle$ be an I-model, and let $\mathfrak {M}^{\,\prime }$ = $\langle W,\le ,v ^+, v^-\rangle$, where the functions $v^+$ and $v^-$ from $\textsf{Prop}$ into subsets of *W* are defined by requiring for every $\alpha \in W$ and $p\in \textsf{Prop}$:

- $$\alpha \in v(p)$$ iff $$\alpha \in v^+(p)$$;
- $$\alpha \in v(p')$$ iff $$\alpha \in v^-(p)$$.

Clearly, $\mathfrak {M}^{\,\prime }$ is a CE-model. For every $\mathcal {L}^{CE}$-formula *A* and $\alpha \in W$,

1. $${\mathfrak M}^{\,\prime }, \alpha \models ^+ A \text{ iff } {\,\mathfrak M}, \alpha \models \tau (A)$$,
2. $${\mathfrak M}^{\,\prime }, \alpha \models ^- A \text{ iff } {\,\mathfrak M}, \alpha \models \tau ({\sim }A)$$.

### Proof

By simultaneous induction on the construction of *A*. $\square$

### Theorem 5

(Semantical embedding) For any set of $\mathcal{L}^{{CE}}$-formulas $\Gamma \cup \{ A \}$, $\Gamma \models _{\textsf{CE}}$ *A* iff ${\tau (\Gamma )\models _{\textsf{Int}^+} \! \tau (A)}$.

### Proof

By contraposition. Suppose $\Gamma \not \models _{\textsf{CE}} A$. Then there exists a CE-model $\mathfrak {M}^{\,\prime } =$ $\langle W, \le , v^+, v^-\rangle$ and $\alpha \in W$ such that $\mathfrak {M}^{\,\prime }, \alpha \not \models A$ but $\mathfrak {M}^{\,\prime }, \alpha \models B$ for every $B \in \Gamma$. By Lemma 3, there is an I-model $\mathfrak M =$ $\langle W, \le , v\rangle$ such that $\mathfrak {M}, \alpha \not \models _{\textsf{Int}^+} \tau (A)$ but $\mathfrak {M}, \alpha \models _{\textsf{Int}^+} \tau (B)$ for every $B \in \Gamma$. The direction from left to right is analogous. $\square$

### Theorem 6

(Completeness of $\textsf{LCE}$) For any finite set of $\mathcal{L}^{{CE}}$-formulas $\Gamma \cup \{ A \}$, $\textsf{LCE}$ $\vdash \Gamma \Rightarrow A$ iff $\Gamma \models _{\textsf{CE}} A$.

### Proof

$\textsf{LCE}$ $\vdash \Gamma \Rightarrow A$ iff (by Theorem 2) $\textsf{LInt}^+ \vdash \tau (\Gamma )\Rightarrow \tau (A)$ iff (by soundness and completeness of $\textsf{Int}^+$ with respect to the class of all I-models) $\tau (\Gamma ) \models _{\textsf{Int}^+}$ $\tau (A)$ iff (by Theorem 5) $\Gamma \models _{\textsf{CE}} A$. $\square$

Finally, as another important feature of $\textsf{LCE}$, we may note a weak replacement property. If *A* and *B* are $\mathcal{L}^{{CE}}$-formulas, let ${A\Leftrightarrow B}$ abbreviate the four sequents $A\Rightarrow B$, $B\Rightarrow A$, ${\sim }A\Rightarrow {\sim }B$ and ${\sim }B\Rightarrow {\sim }A$. Say that ${A\Leftrightarrow B}$ is provable in $\textsf{LCE}$ if $A\Rightarrow B$, $B\Rightarrow A$, ${\sim }A\Rightarrow {\sim }B$ and ${\sim }B\Rightarrow {\sim }A$ are provable in $\textsf{LCE}$. Let the degree of *A*, *d*(*A*), be the number of occurrences of connectives in *A*.

### Theorem 7

(Weak replacement) If ${A\!\Leftrightarrow \!B}$ is provable in $\textsf{LCE}$, then so is ${C_A\!\Leftrightarrow \!C_B}$.

### Proof

The proof is by induction on $l = d(C_A) - d(A)$. If $l = 0$, the proof is trivial because then *C* $=$ *p* for some propositional variable *p*. Assume that the claim holds for every $l \le m$, and let $l = m +1$. If $C_A$ is a formula ${\sim }D$, assume that $d(D_A) \le l$ and ${A\!\Leftrightarrow \!B}$ is provable in $\textsf{LCE}$. Then, by the induction hypothesis, $D_ A \Leftrightarrow D_B$ is provable in $\textsf{LCE}$ and hence the following sequents are provable in $\textsf{LCE}$: $D_A \Rightarrow D_B$, $D_B \Rightarrow D_A$, ${\sim }D_A \Rightarrow {\sim }D_B$, ${\sim }D_B \Rightarrow {\sim }D_A$. With (${\sim }{\sim }$l) and (${\sim }{\sim }$r), also ${\sim }{\sim }D_A \Rightarrow {\sim }{\sim }D_B$, ${\sim }{\sim }D_B \Rightarrow {\sim }{\sim }D_A$ are provable in $\textsf{LCE}$, and thus ${C_A\!\Leftrightarrow \!C_B}$ is provable in $\textsf{LCE}$. For the cases $C_A = (D\sharp E)$ and $\sharp \in \{\wedge , \vee , \nleftarrow \}$, we here consider the case of $\sharp$ $=$ $\nleftarrow$. We apply the induction hypothesis and obtain the following derivations:$\square$

## Bi-connexivity for Implication and Exclusion

### Bi-connexive Logics

There is not a single notion of duality. As Atiyah (2007) put it:

Fundamentally, duality gives two different points of view of looking at the same object. There are many things that have two different points of view and in principle they are all dualities.

As a corollary to the principle of duality mentioned above, Church (1956, p. 108), for example, states the *special principle of duality for implication*, which relates duality to order-inversion, as in order theory: “If $\vdash A \supset B$, if $A_1$ and $B_1$ are duals of *A* and *B* respectively, then $\vdash B_1 \supset A_1$”. In terms of derivability, the notion of duality applied is that if $A \vdash B$, then $B^* \vdash A^*$, where $^*$ is the operation that replaces every occurrence of a connective in a formula by its dual.

The co-implication connective, $\not \prec$, in dual intuitionistic logic as well as in the logic obtained by combing intuitionistic and dual intuitionistic logic, known as *Heyting-Brouwer logic*, HB, see (Rauszer, 1974a, b, 1977; Goré & Shillito, 2020; Drobyshevich et al., 2022) or *bi-intuitionistic logic*, BiInt, see (Goré, 2000) has been referred to as a *dual* of intuitionistic implication. A different point of view for dualization is available in logical bilateralism, when the above distinction is drawn between support of truth and support of falsity, especially when the conditions (*con*) and (*com*) are not imposed so that support of truth and support of falsity are completely independent of each other and on a par at the level of valuations. Then, the co-implication, denoted by , of the system 2Int introduced in (Wansing, 2016a) can be seen as being a dual of the intuitionistic conditional in another sense. Whereas the intuitionistic conditional internalizes (or expresses) the preservation of support of truth from the premises to the conclusion of an inference in the object language, the co-implication of 2Int, internalizes the preservation of support of falsity.

There is thus more than one way of dualizing the constructive implication of intuitionistic logic; and there is also more than one way of dualizing the connexive implication of C. A ‘bi-connexive’ logic, 2C, has been defined in (Wansing, 2016b). In addition to a constructive and connexive implication, it contains in its language a constructive co-implication, denoted by , that is dual to the connexive implication in another sense. The logic 2C is bi-lateral insofar as its natural deduction proof system, N2C, makes use of two derivability relations, namely a provability relation that captures preservation of support of truth from the premises to the conclusion of an inference, and a relation of dual provability (refutability) that captures the preservation of support of falsity from the premises to the conclusion of an inference. As in the case of 2Int, the constructive implication internalizes the provability relation into the object language and the constructive co-implication of 2C internalizes the refutability relation into the object language. The logics 2Int and 2C differ, however, in their understanding of the refutation of implications and proof of co-implications. The encoding of derivations in 2C by typed $\lambda$-terms makes use of a two-sorted typed $\lambda$-calculus that encodes both the introduction of a conditional and the introduction of a co-implication by $\lambda$-abstraction. In that sense, the co-implication of 2C *is* a conditional, its connexive version may be seen as a connexive co-implication, and 2C may be regarded as a bi-connexive logic.

Models for 2C are like models for C, except that there are also the following evaluation clauses for formulas : 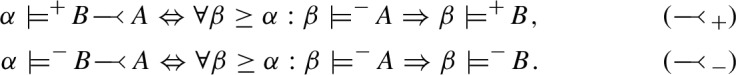


A sequent calculus for L2C is obtained from the sequent calculus LCE by adding the sequent rules for connexive implication from C (see Sect. 6.2) and replacing the rules for $\nleftarrow$ by the following left and right introduction rules:In the bi-lateral natural deduction proof system N2C for 2C, the following dual versions of Aristotle’s and Boethius’ theses are *refutable*:

dAT
,
dAT$$'$$
,
dBT
,
dBT$$'$$
.

If *B* is one of the above formulas, this means that the sequent $\varnothing \Rightarrow {\sim }B$ is provable in LCE. Moreover,  is not refutable in N2C. In (Wansing & Omori, 2024) it has been suggested to call a logic ‘dually connexive’ if its co-implication connective satisfies non-symmetry of co-implication and if it validates dAT–dBT$'$ for the entailment relation coinciding with the derivability relation internalized by co-implication.

Due to the presence of the strong negation from C, implication and co-implication in 2C are interdefinable, and due to the presence of the falsum constant $\bot$, 2C is definitionally equivalent with C$^\bot$.^6^

A “bi-intuitionistic connexive logic”, BCL, has been studied in (Kamide & Wansing, 2016). The system BCL is an expansion of C$^\bot$ by a ‘connexive’ variant of the co-implication used in dual intuitionistic logic and in HB. The logic BCL is also called ‘connexive Heyting-Brouwer Logic’. Since in its Kripke semantics, the co-implication of Heyting-Brouwer logic and of BCL (denoted there also by ) is defined semantically in terms of a backward looking existential quantifier over states, it is hardly a conditional. Nevertheless,  in BCL can also be considered dual to the connexive (C-like) implication $\rightarrow$, since their semantic definitions are obtainable from each other by a throughout interchanging between dual notions in the metalanguage: $\forall$ and $\exists$, $\wedge$ and $\vee$, as well as by reversing the accessibility relation. BCL is a system with a connexive implication and a co-implication connective whose falsity condition has been modified to the effect that the following equivalence is provable in the sequent calculus for BCL:

.

Thus, as we have seen, there can be several ways to combine within a joint framework a connexive implication with a connective that is in some sense dual to it. In the next subsection, we show how this kind of combination can be realized by involving the connexive exclusion introduced in this paper.

### Uniting C and CE: The Bi-connexive Logic bCE

An often considered example of a case speaking against expressive parsimony is the Sheffer stroke, ‘$\mid$’, definable in classical logic by setting ${A\!\mid \!B:= {-}(A\wedge B)}$. Although the set $\{\mid \}$ is functionally complete for Boolean propositional logic, the logic is usually presented by using the set of connectives ${\{{-},\wedge ,\vee ,\supset \}}$. The choice of connectives matters, and it maters not only for practical reasons such as succinctness, readability, or processability of notation. The choice of connectives can be a source of inspiration for further (in particular, philosophical) questions and investigations, and it may have repercussions on, for example, the axiomatization of a logic, cf. (Humberstone, 2004; Omori & Skurt, 2020).

The definability of $\rightarrow$ in CE and $\nleftarrow$ in C does not speak against having available both implication and exclusion as primitive connectives. If it makes sense to distinguish between classical logic based on $\{\mid \}$ versus classical logic based on $\{{-}, \wedge , \vee , \supset \}$, it makes sense to distinguish not only between C and CE, but also between them and their combination, which one may refer to as bCE because implication and exclusion can be seen as two separate concepts in their own right.

Let us consider the united language $\mathcal{L}^{bCE}$:

$$\begin{aligned} A {:}{:} = p \mid (A\wedge A) \mid (A \vee A) \mid (A \rightarrow A) \mid A \nleftarrow A \mid {\sim }A. \end{aligned}$$

To obtain bCE-models one can simply add the conditions ($\nleftarrow _+$) and ($\nleftarrow _+$) to the definition of C-models. Fortunately, we do not need any manipulation of the heredity conditions, since both C-models and CE-models accept FH$^{+}$ and FH$^{-}$. Validity and entailment for bCE-models are still defined as in Definition 4 by replacing $\mathfrak {M}^{I}$ with $\mathfrak {M}^{bCE}$.

Most importantly, neither $\rightarrow$ nor $\nleftarrow$ are contrapositive in bCE-models, in the sense that neither $A \rightarrow B \models {\sim }B \rightarrow {\sim }A$ nor $A \nleftarrow B \models {\sim }B \nleftarrow {\sim }A$ hold (nor are any of their variations given double negations, such as $A \rightarrow {\sim }B \models B \rightarrow {\sim }A$). Otherwise, both implication and exclusion would be symmetric, thus collapsing respectively to biconditional and incompatibility. Indeed, it is not difficult to check that the following chains of entailments would hold, provided that $A \rightarrow {\sim }B \models B \rightarrow {\sim }A$ and ${\sim }B \nleftarrow A \models {\sim }A \nleftarrow B$ were valid:

$$\begin{aligned} A\nleftarrow B \models B\rightarrow {\sim }A \models A \rightarrow {\sim } B \models B\nleftarrow A;\\ A\rightarrow B \models {\sim }B\nleftarrow A \models {\sim }A \nleftarrow B \models B\rightarrow A. \end{aligned}$$

Thus, employing a non-contraposable logical framework turns out to be essential for explicating the operation of connexive exclusion, and it is noteworthy that the implication of the connexive logic C (in contrast to classical and intuitionistic implication) is most suitable for this purpose.

To obtain a binary bi-connexive consequence system $\mathsf {bCE_c}$ one has to add to $\mathsf {CE_c}$ the following axioms and a rule governing $\rightarrow$:
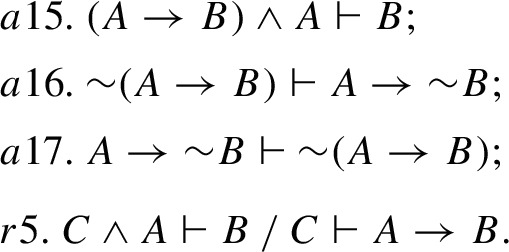


It is an easy exercise to show that $\mathsf {bCE_c}$ is sound with respect to the class of all bCE-models. To establish completeness, we involve the notion of a bCE-theory, which is defined exactly as that one of a CE-theory, but in the language $\mathcal{L}^{bCE}$ and with respect to the relation $\vdash _{\mathsf {bCE_c}}$. Lemma 1 holds for the bCE-theories, and the canonical bCE-model is defined as in Lemma 2*mutatis mutandis*. Now, to see that the canonical bCE-model so defined is indeed a bCE-model, one has to extend the proof of Lemma 2 with the case for $\rightarrow$.

#### Proof

Assume $A \rightarrow B \in t$. Consider an arbitrary $t^\prime \supseteq t$, such that $A \in t^\prime$. Then, by transitivity of $\supseteq$, $A \rightarrow B \in t^\prime$. By definition of a bCE-theory, $A \wedge (A \rightarrow B) \in t^\prime$, and by *a*15, $B \in t^\prime$. For the converse, assume $A \rightarrow B \notin t$. Consider the theory $t + A$. First, $B \notin t + A$. Indeed, if $B \in t + A$, then there exists some finite set {$C_1, \ldots , C_n\} \subseteq t$, such that $(C_1 \wedge \ldots \wedge C_n) \wedge A\vdash _{\mathsf {bCE_c}} B$. By *r*5, $C_1 \wedge \ldots \wedge C_n \vdash _{\mathsf {bCE_c}} A \rightarrow B$, and hence, $A\rightarrow B \in t$, a contradiction. Second, by Lemma 1.2 adjusted for bCE-theories, $t + A$ can be extended to a prime bCE-theory, such that *B* still does not belong to it, and so, is non-trivial. Hence, $A \rightarrow B \in t \Leftrightarrow \forall t^\prime \supseteq t: A\in t^\prime \Rightarrow B \in t^\prime$.

Next, assume ${\sim }(A\rightarrow B) \in t$. Then, by *a*16, $A\rightarrow {\sim }B \in t$. Consider an arbitrary $t^\prime \supseteq t$, such that $A\in t^\prime$. Obviously, $A\rightarrow {\sim }B \in t^\prime$, and hence, $A \wedge (A\rightarrow {\sim }B) \in t^\prime$. Then, by *a*15, $B\in t^\prime$. Conversely, assume ${\sim }(A\rightarrow B) \notin t$, and consider the theory $t + A$. By the same line of reasoning as above, and by using *a*17 and *r*5, we can show that ${\sim }B \notin t + A$. By Lemma 1.2 adjusted for bCE-theories, there is a prime bCE-theory $t^\prime \supseteq t$, such that ${\sim }B\notin t^\prime$. Hence, ${\sim }(A \rightarrow B) \in t \Leftrightarrow \forall t^\prime \supseteq t: A\in t^\prime \Rightarrow {\sim }B \in t^\prime$. $\square$

Now, when Lemma 2 is appropriately extended to bCE-theories, Theorem 1, *mutatis mutandis*, gives us the completeness of $\mathsf {bCE_c}$ with respect to bCE-models.

Remarkably, in the system $\mathsf {bCE_c}$ certain consequences are derivable, which reflect the interdefinability of $\rightarrow$ and $\nleftarrow$. Namely, we almost immediately get the following two simple derivations:
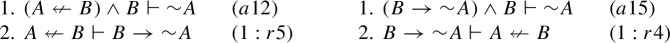


that establish the interderivability of $A \nleftarrow B$ and $B \rightarrow {\sim }A$, as it should be according to the semantic justification of connexive implication in C-models and connexive exclusion in CE-models. And in the same way we obtain the interderivability of $A \rightarrow B$ and ${\sim }B \nleftarrow A$, which implements the main idea of Definition 3.

The sequent calculus LbCE is obtained from LCE by adding the following sequent rules for connexive implication:In LbCE the sequents $A \Rightarrow A$ are derivable for any $\mathcal {L}^{bCE}$-formula *A*.

The translation $\tau$ from Definition 8 can be extended to a mapping from $\mathcal {L}^{bCE}$ into $\mathcal {L}^+$ based on $\textsf{Prop} \cup \textsf{Prop}'$ by adding the following clauses:

$$\begin{aligned} \tau (A \rightarrow B):= \tau (A) \rightarrow \tau (B) \text{ and } \tau ({\sim }(A \rightarrow B)):= \tau (A) \rightarrow \tau ({\sim }B). \end{aligned}$$

This gives a syntactical embedding of $\textsf{LbCE}$ into $\textsf{LInt}^+$:

1. $$\textsf{LbCE} \vdash { {\Gamma } \Rightarrow A }$$ iff  $$\mathsf {LInt^+}$$ $$\vdash { \tau ({\Gamma }) \Rightarrow \tau (A) }$$,
2. $$\textsf{LbCE}-\{{\mathrm{(cut)}}\} \vdash { {\Gamma } \Rightarrow A }$$ iff  $$\mathsf {LInt^+}\!-\!\{\mathrm{(cut)}\}$$ $$\vdash { \tau ({\Gamma }) \Rightarrow \tau (A) }$$.

The admissibility of (cut) in cut-free $\textsf{LbCE}$ then follows as in the case of $\textsf{LCE}$, and as a corollary to the admissibility of (cut) we obtain the paraconsistency of $\textsf{LbCE}$ with respect to ${\sim }$ and $\wedge$, its paracompleteness with respect to ${\sim }$ and $\vee$, and the decidability of $\textsf{LbCE}$. Moreover, it can show that the extended translation $\tau$ gives rise to a semantical embedding of $\textsf{bCE}$ into $\textsf{Int}^+$ and thereby that $\textsf{LbCE}$ is complete with respect to the class of all bCE-models. For any finite set of $\mathcal{L}^{{bCE}}$-formulas $\Gamma \cup \{ A \}$, $\textsf{LbCE}$ $\vdash \Gamma \Rightarrow A$ iff $\Gamma \models _{\textsf{bCE}} A$.

Also, the weak replacement result for $\textsf{LCE}$ extends to $\textsf{LbCE}$. The following four derivations in LbCE show that $A \rightarrow B$ and ${\sim }B \nleftarrow A$ are replaceable for each other in LbCE:

## Conclusion and Future Prospects

Starting from the notion of duality of connectives as defined by Church (1956) for classical logic, we arrived at the concept of exclusion as a dual to implication in a constructive setting. We have pointed out that, within both classical and intuitionistic logic, this notion lacks the property of connexivity, which can be naturally expressed by some principles analogous to Aristotle’s and Boethius’ theses for connexive implication. We provided an intuitively plausible semantic justification for the idea of connexive exclusion based on a certain dualization of a semantic framework for the logic of connexive implication C. The propositional logic we arrived at is definitionally equivalent with the constructive, paraconsistent, paracomplete, and negation inconsistent connexive logic C. We presented the logic of connexive exclusion, CE, i.e., the logic with the connexive exclusion connective as a primitive operation, as a binary Fmla-Fmla consequence system, $\mathsf {CE_c}$, and as a sequent calculus, $\textsf{LCE}$, and showed completeness with respect to the class of all CE-models. As in the case of the sequent calculus for C, the sequent system $\textsf{LCE}$ has nice proof-theoretic properties. Moreover, we suggested to combine C and CE, i.e., to consider both the connexive implication and the connexive exclusion connective as primitive.

As already observed, the connexive implication in C lacks the property of contraposition, and accordingly, the connective of connexive exclusion introduced and studied in this paper lacks the respective property as well. Sometimes it is considered to be desirable for the implication to be contraposable. Nonetheless, for a number of logics, in particular those dealing with the constructive notion of falsity and strong Nelson-type negation, it is very important not to validate generally the property of contraposition in order to avoid collapsing to classical logic. Also, contraposition fails for some context-dependent conditionals, and for some types of consequential implication considered by Pizzi (2018). However, there are various systems of connexive logic, the implication of which is contraposable, see, e.g., (Wansing, 2023a). It is clear that if one will build on such systems rather than on C, the corresponding connective of connexive exclusion will differ from the one developed in this paper. But this is a matter for a broader project, which we leave for future work.

The logic of connexive exclusion devised in this paper presents another, and we believe, interesting case of a logic belonging to the class of negation inconsistent nontrivial logics. Such logics are justified and propagated in (Wansing, 2023b), where it is argued that it is theoretically rational to believe not only that there exist interesting or important non-trivial negation inconsistent theories but also that there exist interesting or important non-trivial negation inconsistent logics. Moreover, a number of such logics are listed and it is emphasized that they are obtained by considerations that are completely independent from the desire to present examples of negation inconsistent nontrivial logics.

For example, it turns out that any connexive logic which retains usual properties of (extensional) conjunction and disjunction, such as conjunction elimination and disjunction introduction, together with the De Morgan laws, must be negation inconsistent. Yet, in a paraconsistent setting this by no means makes such logics “absurd” from an informal or philosophical perspective. On the contrary, the negation inconsistency of these logics gives rise to a number of challenging problems such as the classification of their provable contradictions, cf. (Niki & Wansing, 2023; Niki, 2023), their use in theory formation, and their role in the methodology and philosophy of logic.

## Footnotes

1 The connective of cotenability is also widely used under the name “fusion”, in particular in various relevance logics, see, e.g., (Dunn & Restall, 2002, pp. 12, 60, ff.).

2 Note that Definitions 2 and 3 allow for mutual definability of cotenability and exclusion. (We thank Claudio Pizzi for this observation.)

3 Here we use a not quite conventional symbol for intuitionistic implication to distinguish it from material implication ($\supset$) and connexive implication ($\rightarrow$).

4 It should be observed that in the literature slightly different definitions of $\models ^+$ and $\models ^-$ for $\nprec$ are widespread, according to which the right-hand side in ($\nprec _+$) would be $\exists \beta \ge \alpha : {\beta }\models ^+\! A \text{ and } {\beta }\models ^-\! B$, and in ($\nprec _-$) it would be $\forall \beta \ge \alpha : {\beta }\models ^+\! A \Rightarrow {\beta }\models ^+\! B$. Such definitions actually make the dual intuitionistic implication $A \nprec B$ dual not to implication $A \succ B$, but to its converse $B\succ A$. There are some historical and algebraic reasons for this, see a discussion of this issue in (Schroeder-Heister, 2009, pp. 1084–1085), (Shramko, 2016, p. 252).

5 The plausibility of the converses of BT and BT$^\prime$ has been contested, for instance by McCall (2012), and a referee expressed the view that the property of being hyperconnexive is anomalous because it has the consequence that “Aristotle’s Thesis is connexively equivalent to Identity, which amounts to a form of partial trivialization." However, the connexive equivalence of AT with ${{\sim }A\rightarrow {\sim }A}$, and in C even with ${A\rightarrow A}$, could also be seen as a confirmation of the view that the validity of AT is desirable.

6 Note that ${\sim }\lnot A$ is provable in C$^{\bot }$ and 2C for any formula *A*, if $\lnot A$ is defined as $A\rightarrow \bot$. The provability of ${\sim }(A \rightarrow \bot )$ in the logic dLP, (dialetheic LP), has been observed in (Omori, 2016, Remark 13) and its provability in 2C has been pointed out in (Wansing, 2016b, Sect. 5).
